# Targeting ACAT1 in cancer: from threat to treatment

**DOI:** 10.3389/fonc.2024.1395192

**Published:** 2024-04-24

**Authors:** Tie Sun, Xuan Xiao

**Affiliations:** Department of Thyroid and Breast Surgery, People’s Hospital of China Medical University (Liaoning Provincial People’s Hospital), Shenyang, China

**Keywords:** metabolism, immunity, ACAT (acyl-CoA:cholesterol acyltransferase), cancer, cholesterol

## Abstract

Altered cholesterol metabolism has been identified as a critical feature of cancers. Cholesterol functions as the main component of cell membrane, cholesterol and is required for sustaining membrane integrity and mediating signaling transduction for cell survival. The intracellular level of cholesterol is dynamically regulated. Excessive cholesterol could be converted to less toxic cholesteryl esters by acyl-coenzyme A:cholesterol acyltransferases (ACATs). While ACAT2 has limited value in cancers, ACAT1 has been found to be widely participated in tumor initiation and progression. Moreover, due to the important role of cholesterol metabolism in immune function, ACAT1 is also essential for regulating anti-tumor immunity. ACAT1 inhibition may be exploited as a potential strategy to enhance the anti-tumor immunity and eliminate tumors. Herein, a comprehensive understanding of the role of ACAT1 in tumor development and anti-tumor immunity may provide new insights for anti-tumor strategies.

## Introduction

A hallmark of cancer is the deregulated metabolism (1). As an integral component of cell membrane, cholesterol is crucial for maintaining membrane integrity and signaling transduction for cell survival (2). Besides, cholesterol also participates in the regulation of multiple biological processes, including lipid metabolism, inflammation, apoptosis, and cell survival (3–5). Cholesterol-derived metabolites exert a wide variety of biological effects in tumor development and anti-tumor immunity responses (6). As fast-proliferating cells, tumor cells rely on cholesterol for membrane biogenesis and various biological processes (7). Therefore, targeting cholesterol metabolism may provide novel therapeutic strategies for cancer management.

Intracellular cholesterol is dynamically transported for maintaining membrane integrity (8). Excessive cholesterol is either exported by ATP-binding cassette proteins, or converted to less toxic cholesteryl esters by acyl-coenzyme A: cholesterol acyltransferases (ACATs) to store in the form of lipid droplets or lipoproteins (3). ACATs belong to membrane-bound O-acyltransferase family, are composed of two enzymes localizing in the mitochondria and cytoplasm, respectively (9). ACAT1 and ACAT2 catalyze acyl transfer from acyl-coenzyme A (CoA) to cholesterol and produce cholesterol esters that are used for storage and intercellular transport of sterol, which is important for cellular cholesterol homeostasis (10). ACAT1 is expressed in nucleated eukaryotic cells and its products are incorporated into lipid droplets (LDs) in the cytoplasm (11). ACAT2 is primarily expressed in intestinal epithelial cells and hepatocytes, and its products are incorporated into lipoproteins in the endoplasmic reticulum (12). While ACAT2 has limited value in tumors, ACAT1 has been found to be implicated in tumor occurrence and development. Recent studies introduced the complex role of ACAT1 in tumor development and anti-tumor immunity, which may provide new insights for anti-tumor strategies.

## ACAT1 structure, regulation, and function

**Figure 1:**
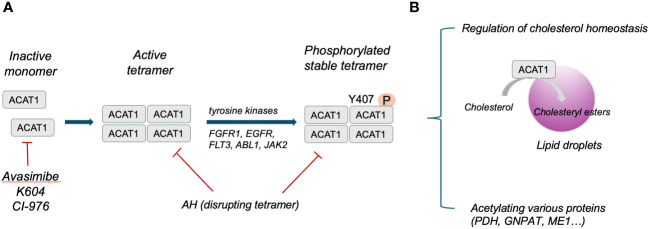
**(A)**, Schematic illustration of ACAT1 structure. **(B)**, Primary function of ACAT1 in cells.

Structure analysis has identified human ACAT1 as a tetramer with two homodimers (13). Each monomer is composed of nine transmembrane segments, which enclose a cytosolic tunnel and a transmembrane tunnel that converge at the predicted catalytic site (14). ACAT1 tetramers, but not monomers, are phosphorylated and stabilized by enhanced Y407 phosphorylation observed in multiple human cancer cells. It has also been indicated that CoA could enter through the cytosolic tunnel, while cholesterol enters via the transmembrane tunnel (13). The structure of ACAT1 has been deciphered previously (13, 14). ACAT1 exerts its catalytic role in ketolysis, ketogenesis, fatty acid oxidation and isoleucine degradation (12). ACAT1 senses free cholesterol by its allosteric site. ACAT1 cannot exert its catalytic role for esterification with high efficiency under low cholesterol concentrations, whereas high amounts of cholesterol could facilitate esterification allosterically under high cholesterol concentrations (13). Herein, ACAT1 activity is determined by the level of free cholesterol to regulate cholesterol homeostasis of the endoplasmic reticulum (15). In addition to mediating cholesterol homeostasis, ACAT1 exerts its acetyltransferase activity capable of specifically acetylating various enzymes. For instance, ACAT1 regulates pyruvate dehydrogenase complex (PDC) by acetylating pyruvate dehydrogenase (PDH) and PDH phosphatase to promote glycolysis (16). ACAT1-mediated K128 acetylation of GNPAT could protect FASN from degradation and promote lipid metabolism (17). ACAT1-mediated K337 acetylation of ME1 dimerize and activate ME1 to regulate NADPH generation and lipid metabolism (18). The structure and function of ACAT1 has been illustrated in **Figure 1**.

## ACAT1 and immunity

**Figure 2:**
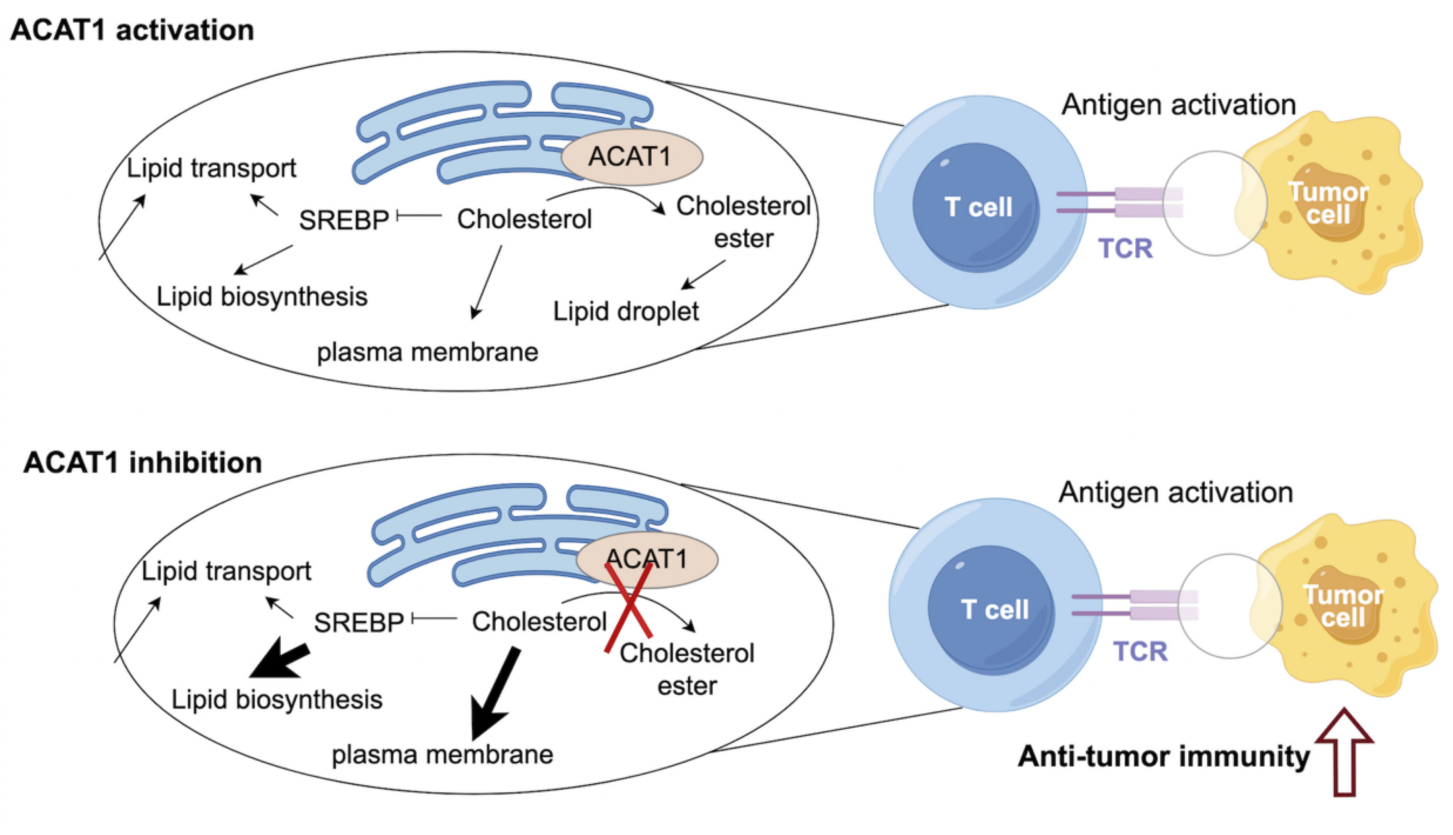
ACAT1 is a potential target to enhance the anti-tumor strategy.

Cholesterol metabolism has been identified to be essential for immune function (4, 19). Cholesterol biosynthesis is critical for T cell growth, activation, and anti-tumor function (4, 20, 21). It has been demonstrated that elevated levels of cholesterol in T cells could boost the anti-tumor immunity of T cells (22). CD8+ T cells play an essential role in anti-tumor immunity, but their function is always abrogated in the context of cancers (23). Therefore, remodeling the anti-tumor ability of CD8+ T cells is a key strategy for improving the efficacy of immunotherapy. ACAT1 inhibition could impair cholesterol esterification, therefore potentiating anti-tumor effect and strengthening cell proliferation of CD8+ T cells (22). Mechanistically, elevated cholesterol level of CD8+ T cells could enhance T-cell receptor clustering and signaling. ACAT1-deleted CD8+ T cells exhibited impaired tumor growth and metastasis of melanoma (22). An avasimibe-induced inhibition of cholesterol esterification has been shown to improve the antitumor response of CD8+ T cells in mice (24). Avasimibe exerted significant anti-tumor effect. Moreover, avasimibe combined with PD-1 inhibitor exhibited greater anti-tumor capabilities compared with PD-1 inhibitor alone. Avasimibe could be restrained on the T cell surface to induce rapid T cell receptor clustering and sustaine T cell activation (25). In addition, paclitaxel and immunoadjuvant αGC were co-encapsulated in liposomes modified with pH sensitive TH peptide (PTX/αGC-TH-Lip). Avasimibe could elevate the level of free cholesterol and relieve the inhibition of CD8+ T cells resulted from PTX/αGC-TH-Lip. The combination of avasimibe and PTX/αGC-TH-Lip could enhance immune responses and cytotoxic effects in xenografts of melanoma, which is a potential strategy to improve the anti-tumor effects of immune-chemotherapy (26) (**Figure 2**).

## ACAT1 in different tumor types

The role of metabolic enzymes in various biological processes has been gradually discovered. It has been well-established that multiple metabolic enzymes could participate in epigenetic remodeling by providing substrates such as acetyl-CoA (27, 28). Considering the catalytic role of ACAT1 in mediating acyl transfer from CoA to cholesterol, ACAT1 may exert complex and dynamic role in tumorigenesis and progression. Mounting evidence has elucidated the significance of ACAT1 in multiple malignances. Here, we summarized the role of ACAT1 in the contexts of different cancers.

## ACAT1 and colorectal cancer

Colorectal cancer (CRC) is the third most diagnosed tumor worldwide (29). 25% of newly diagnosed CRC patients are diagnosed at the metastatic stage (30). The crosstalk between cholesterol metabolism and CRC has been under vast investigations. An elevated cholesterol level could accelerate CRC progression by activating β-catenin oncogenic signaling pathway (29). Specific liver metastases of CRC display an aberration of cholesterol biosynthesis (31). Thus, aberrant cholesterol metabolism is a hallmark of CRC, which may be exploited as potential therapeutic targets.

Numerous evidence illustrated that various molecular mechanisms are engaged in the tumorigenesis and development of CRC (32). The cytoplasmic form of malic enzymes (ME), ME1, has been identified as a primary source of NADPH for lipogenesis and glutamine biosynthesis. In CRC cells, depletion of ACAT1 could dramatically impair ME1 acetylation without influencing its protein level, whereas ACAT1 overexpression exerts the opposite effect. Moreover, ACAT1 overexpression enhances ME1 dimerization whereas deletion of ACAT1 expression impair ME1 dimerization. This ACAT1-mediated K337 acetylation positively regulates ME1 dimerization. PGAM5, a mitochondrial serine/threonine phosphatase, could dephosphorylate ME1 at S336, further promoting acetylation by ACAT1 at the adjacent K337. ACAT1-mediated ME1 K337 acetylation could enhance NADPH generation, lipogenesis, and CRC tumorigenesis (18). β-hydroxybutyrate (BHB) was previously identified as an oncogenic metabolite of CRC, which was also found to be elevated in CRC tissues. BHB has been found to promote CRC progression by ACAT1 by mediating acetylation of isocitrate dehydrogenase 1. ACAT1 abrogation could impair CRC tumorigenesis and abrogate the tumorigenic effects of BHB (33, 34). Small molecule inhibitors that target ACAT1-mediated ME1 acetylation may be a potential anti-tumor strategy for CRC patients. Besides, the development of CRC is correlated with hyperinsulinemia. Insulin-induced tumor progression of CRC is regulated by ACAT1. Insulin enhanced CRC development by upregulating ACAT1, which can be exploited as a promising therapeutic target for CRC (35). Collectively, the understanding of CRC biological features associated with ACAT1 may be beneficial for diagnosis and treatment of CRC in the clinical settings (36).

## ACAT1 and hepatocellular carcinoma

Liver cancer is the sixth most diagnosed cancer worldwide (29). Notably, it is highly refractory to most chemotherapeutic regimens. Hepatocellular carcinoma (HCC) is the most common type of liver cancer (37). In HCC cells, ACAT1 could stabilize and dimerize glyceronephosphate O-acyltransferase (GNPAT), a rate-limiting enzyme in plasmalogen synthesis and lipogenesis, by acetylation at K128. Precisely, ACAT1-mediated GNPAT acetylation could inhibit GNPAT degradation by repressing TRIM21-mediated GNPAT ubiquitination, ultimately promoting tumor growth in HCC xenografts. ACAT1 overexpression enhanced tumor growth in HCC xenografts and GNPAT deletion could attenuate ACAT1-induced HCC growth, and ACAT1 overexpression with GNPAT inhibition diminish fatty acid synthesis and lipogenesis. Combination treatment of ACAT1 inhibitor and sorafenib could significantly inhibit tumor growth in HCC xenografts, indicating that pharmaceutical inhibition of ACAT1 could be a promising target in anti-HCC strategy (17). In HEK293 cells, ACAT1 has been identified as a substrate of E3 ubiquitin ligase UBE3A/E6AP. High-fat diet could downregulate UBE3A expression, while UBE3A overexpression could lead to decreased ACAT1 protein level (38). Future studies may require combination regimens that include systemic therapies and molecularly targeted treatments, such as ACAT1.

## ACAT1 and glioblastoma

Glioblastoma (GBM) are the most frequently diagnosed malignant primary brain tumors that originate from neuroglial progenitor cells (39). Conventional treatment could bring limited improvements in the survival of glioma patients, leading to poor survival outcomes for GBM patients (40). Herein, it is desperately required for effective molecularly targeted therapy to improve prognosis of GBM patients. In GBM patients, ACAT1 has been found to be upregulated and correlated with poor prognosis. Moreover, pharmacological inhibition of ACAT1 in GBM cells demonstrated that ACAT1 is required for GBM proliferation (41). Upon inhibition of mTORC1, ACAT1 could catalyze acetylation of glycine decarboxylase (GLDC), a critical enzyme of glycine metabolism that catalyzes the conversion of glycine into one-carbon units. The acetylation of GLDC at K514 inhibits its enzymatic activity, which promoted K33-linked polyubiquitination at K544 by NF-X1, resulting in GLDC degradation by the proteasomal pathway (42). Acetylation of GLDC at K514 could suppress glycine catabolism, pyrimidines synthesis and GBM development. K604, a potent ACAT1 inhibitor, could impair the proliferation of U251−MG cells and inactivate Akt signaling pathway in GBM cells (43). Avasimibe, another specific inhibitor of ACAT, exerts anti-tumor effect on U87, A172 and GL261 GBM cells. In GBM cell lines, avasimibe could inhibit the expression of ACAT1 and biosynthesis of cholesterol ester. Moreover, avasimibe could impair the proliferation of GBM cells resulted from caspase-8 and caspase-3 activation (44). Herein, ACAT1 functions as a novel target for HCC, providing effective assistance to the treatment of GBM.

## ACAT1 and lung cancer

Lung cancer is a heterogenous disease composed of multiple genetic and molecular subtypes, which is still the leading cause of cancer-related death worldwide (45). Considering that molecularly defined subtypes are potentially targetable, novel anti-tumor strategies in for lung cancer are required to be explored. Excessive intracellular cholesterol is catalyzed to cholesteryl esters via ACAT1 and exported via the cholesterol transporter ABCA1. In a cohort of patients with lung adenocarcinoma, ACAT1 has been found to be upregulated, while ABCA1 is downregulated in the lung cancer tissues. In H1299 cells, ACAT1 has been identified as the acetyltransferase of PDHA1 and PDP1. Mechanistically, PDP1 phosphorylation at Y381 recruits ACAT1 and dissociates SIRT3 to promote lysine acetylation of PDP1 and PDHA1. ACAT1 predominantly signals through inhibition of PDC by PDP1 and PDHA acetylation to enhance glycolysis and tumor growth, indicating the ACAT1-PDP1-PDHA axis a promising anti-cancer target (46). lncRNA DARS-AS1 inhibition attenuated non-small cell lung cancer development by activating miR-302a-3p to inhibit ACAT1 expression (47). It has been demonstrated that Kras-specific antigenic peptides in combination of avasimibe could promote CD8+ T cell infiltration and impair lung tumor progression (48). Collectively, ACAT1 may function as a promising therapeutic target for lung cancer.

## ACAT1 and breast cancer

Breast cancer is a heterogeneous malignancy with multiple molecular subtypes based on histological and genomic features (49). Gene expression profiling has proposed four intrinsic molecular subtypes: Luminal A, Luminal B, HER 2+ and basal like (50). It has been demonstrated that ER-negative breast cancer cells display accumulation of LDs, increased LDL uptake, a higher ratio of cholesteryl ester to triacylglycerol, lower cholesterol biosynthesis, increased expression of ACAT1, higher ACAT activity as compared to ER-positive breast cancer cells (51). CP-113,818, a ACAT inhibitor, could inhibit proliferation of breast tumor cells and reduce LDL-mediated proliferation of ER-negative cells. In MDA-MB-231 cells, LDL receptor (LDLR) mRNA could be markedly impaired by ACAT inhibition, indicating that high ACAT1 activity is correlated with higher LDLR expression (52). It has been found that ACAT1 upregulation in breast tumor cells could promote tumor initiation and metastasis, indicating ACAT1 as a metabolic tumor promoter (53). Nuclear receptor subfamily 2 group F member 6 (NR2F6) could transcriptionally activate ACAT1 and enhance the suppressive role of ACAT1-induced METTL3 acetylation on cell migration and invasion of breast cancer (54).

## ACAT1 and leukemia

Leukemia is a heterogeneous malignancy with different genetic, morphologic and molecular feature, which is composed of multiple subtypes including acute myeloid leukemia (AML) and chronic myeloid leukemia (CML) (55). ACAT1 and SIRT3 have been identified as the upstream acetyltransferase and deacetylase of mutant isocitrate dehydrogenase 2 (mIDH2) in AML to regulate K413-acetylation of mIDH2 and inhibit mIDH2 activity (56). Spectromicroscopic analysis in multiple leukemia cell lines has revealed that aberrant accumulation of CE was found in CML (chronic myelogenous leukemia), which may be resulted from altered BCR-ABL kinase activity. Inhibiting cholesterol esterification via avasimibe could significantly suppress CML cell proliferation. Besides, combinational treatment of avasimibe and imatinib brought synergistic effects on blocking cell proliferation in K562R cells (57).

## Implications for targeting ACAT1 in anti-tumor therapy

Targeting ACAT1 has been identified as a potential anti-tumor strategy (58). Avasimibe, also named as avasimin, has been developed as a potent non-specific ACAT1 inhibitor to impair cholesterol esterification in multiple cancer models. *In vitro* studies have elucidated that avasimibe could reduce cholesteryl-ester storage in LDs and increase levels of free cholesterol, leading to cell apoptosis and impaired proliferation (59). ACAT1 inhibitor could also enhance the cytotoxic effects of CD8+ T cells by reprogramming cholesterol metabolism. A combination of avasimin and anti-PD-1 treatment exhibited synergistic cytotoxic effects in suppressing melanoma development (22). Avasimin combined with nanoparticles of doxorubicin showed better anti-tumor efficacy in impairing breast cancer progression (60). The combination of avasimibe and immune-chemotherapy could enhance the anti-tumor effects of immune-chemotherapy. The combinational treatment could increase the level of free cholesterol and relieve the inhibition of CD8+ T cells resulted from PTX/αGC-TH-Lip. The combination of avasimibe and PTX/αGC-TH-Lip could enhance immune responses and cytotoxic effects in xenografts of melanoma, which is a potential strategy to improve the anti-tumor effects of immune-chemotherapy (26). It has been demonstrated that vaccine of Kras-specific antigenic peptides combines with avasimibe could eliminate regulatory T cells and promote CD8+ T cell infiltration (48).

Targeting tetrameric ACAT1 has been proposed as a promising anti-tumor strategy. Arecoline hydrobromide (AH) is a covalent ACAT1 inhibitor that specifically binds to and disrupts ACAT1 tetramers, thereby AH treatment leads to impaired ACAT1 activity. Due to the inhibitory effect of ACAT1 on PDC by acetylating PDH and PDH phosphatase, AH treatment could enhance PDC flux and oxidative phosphorylation to impair tumor growth, making ACAT1 a potential anti-tumor target. Combination treatment of AH with other anti-tumor strategy have shown greater anti-tumor efficacy. In HCC, AH treatment combined with sorafenib could significantly inhibit tumor growth in HCC xenografts (16). CI-976, a small molecule ACAT1 inhibitor, can bind inside the catalytic chamber and blocks the accessibility of the active site residues of ACAT1. CI-976 has been found to reduce atherosclerotic plaques and decrease plasma cholesterol levels in animals fed with high cholesterol diet (61). Another selective ACAT1 inhibitor K604 has been found to impair the proliferation of U251−MG cells and inhibit Akt signaling in glioblastoma cells (43). The current reported ACAT1 inhibitors have been illustrated in **Table 1**. The anti-tumor effect of ACAT1 inhibitors should be further verified in more cancer types *in vitro* and vivo models to explore the cancer types that can be effectively treated with ACAT1 inhibitors. Clinical trials should be accelerated to evaluate the anti-tumor effects of more ACAT1 inhibitors in different cancer types (62).

**Table 1:** Current ACAT1 inhibitors tested in human cancers.

Inhibitors	Disease	Struture	Binding sites	Mechanism of action	Target	Models	Combined therapy	Reference
Arecoline hydrobromide	Hepatocellular carcinoma	–	Cysteine residue (C126) in the ACAT1 catalytic site	Binds to and disrupts ACAT1 tetramers	Cancer cell proliferation and tumor growth	Cellular or mice models	Sorafenib	(16)
Avasimibe	Melanoma, Lewis lung adenocarcinoma	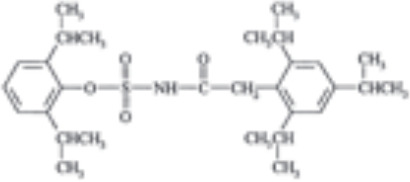	–	A chemical group that conferred ACAT inhibitory properties (the 2,6-diisopropylphenyl moiety)	Tumor growth	Mice models	Anti-PD-1 antibody	(23)
	Chronic myeloid leukemia		–		Tumor growth	Mice models	Imatinib	(57)
K604	Glioblastoma	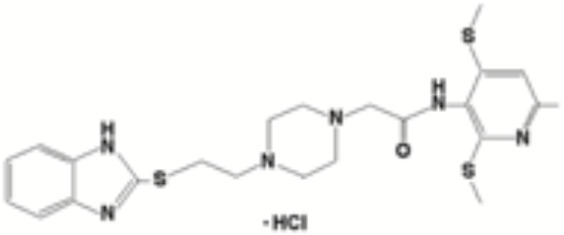	–	–	Cancer cell proliferation	Cellular models	–	(43)

## Author contributions

TS: Writing – original draft, Writing – review & editing. XX: Writing – original draft, Writing – review & editing.
